# Effects of Dark Brooder Rearing and Age on Hypothalamic Vasotocin and Feather Corticosterone Levels in Laying Hens

**DOI:** 10.3389/fvets.2020.00019

**Published:** 2020-01-30

**Authors:** Rebecca E. Nordquist, Elisabeth C. Zeinstra, Alyssa Dougherty, Anja B. Riber

**Affiliations:** ^1^Behaviour and Welfare Research Group, Department of Farm Animal Health, Faculty of Veterinary Medicine, Utrecht University, Utrecht, Netherlands; ^2^UMC Utrecht Brain Center, University Medical Center Utrecht, Utrecht, Netherlands; ^3^Department of Animal Science, Aarhus University, Tjele, Denmark

**Keywords:** dark brooder, feather corticosterone, hypothalamic vasotocin, avian, vasopressin, cortisol

## Abstract

Chickens cannot independently thermoregulate at hatch and lack opportunity to behaviorally thermoregulate with a hen in the egg layer industry, thus barns are heated to thermoneutral temperatures. Dark brooders are low-energy-consuming hot plates, which may be environmentally advantageous while providing welfare-enhancing aspects of maternal care (i.e., shelter and separation of active and inactive individuals). Dark brooder use has been demonstrated to decrease injurious pecking and mortality well into the production period of layers. To further understand hen development around lay onset and effects of dark brooders on the brain and HPA-axis, we examined effects of rearing with dark brooders on expression of vasotocin (AVT) in the hypothalamus and corticosterone (CORT) in the feathers of in total 48 layer Isa Warren hens at 16 w and 28 w of age (*n* = 12 per age and treatment). An age-dependent decreased number of AVT-positive neurons was seen in the medial preoptic area, medial preoptic nucleus, paraventricular nucleus, rostral part (prepeduncular hypothalamus), and lateral preoptic area. Trends to effects of brooder rearing were found in both anteromedial preoptic nucleus and supraoptic nucleus, with dark brooder reared animals showing higher mean counts of AVT-positive neurons in both areas. No interactions between brooder raising and age were observed in AVT-positive neuron count. CORT levels were higher in primary wing feathers from 28 week old hens than in those from 16 week hens. No main effects of rearing with dark brooders or interactions between age and treatment were found on CORT levels. The age-dependent effects seen in the hypothalamus and CORT aids in further understanding of the development of chickens around puberty. The use of brooders tended to increase AVT expression in the anteromedial preoptic nucleus and supraoptic nucleus, an indication that dark brooder rearing may affect physiological responses regulated by these areas. The lack of effect of dark brooders on CORT in feathers is at the least an indication that the use of dark brooders is not stressful; in combination with the benefits of dark brooders on injurious pecking, fearfulness and early mortality, this pleads for the use of dark brooders in on-farm situations.

## Introduction

Chickens (*Gallus gallus domesticus*) hatch with limited ability to produce warmth by thermogenesis (1); upon emergence of thermoregulation at 2 weeks of age, chicks are still unable to maintain their normal body temperature at 21°C or lower ambient temperature (2). Mature thermoregulation is not present until ~4 weeks of age, at least in part due to replacement of downs by feathers. Therefore, chicks' survival in the first weeks depends on their own ability to thermoregulate behaviorally, unless aided artificially.

Under natural conditions, chicks thermoregulate by regularly seeking warmth under a broody hen. In the egg layer industry, adding broody mother hens to flocks of typically several thousand birds in homogenous age groups is not feasible for practical, disease prevention, and economic reasons. Thus, breeding barns are heated to temperatures that allow the chicks to maintain a healthy body temperature, usually starting at 34°C and gradually decreasing to 20–22°C over a period of 4 weeks (3). Barn heating has several drawbacks. First, heating a large space costly in terms of economics and environmental impact. Second, in conventionally heated barns, chicks are not provided with shelter, dark areas, or resting spaces, aspects normally provided by a broody hen which may be important to their development (4–7).

A possible alternative to barn heating is the use of dark brooders: low-energy-consuming hot plates surrounded by horizontal flaps, creating a dark and warm space. Chicks creep under the brooders when they need extra heat, mimicking the use of a broody hen to thermoregulate behaviorally. This allows barn temperature to be reduced, leading to a considerable saving in energy consumption (Unpublished data). Brooders may also have positive effects on welfare problems in the egg layer industry. Chicks bred with broody hens are less fearful than chicks bred without mother hens (8). Likewise, brooders have been shown to reduce the level of fear in chicks (7). Fear is a strong stressor that inhibits the immune system, growth, feed exploitation, and egg production (9), and may also induce or exacerbate feather pecking in laying hens (10). There is strong evidence that the use of dark brooders reduces feather pecking not only during rearing, but as a long-lasting effect into the laying period (6, 7, 11–13).

Dark brooders provide aspects of maternal care that may help chicks in situations where a broody hen is not present (i.e., on farm). Maternal deprivation has been reported to affect the neuro-endocrine response to stress in mammals (14–16). Moreover, an increase in the hypothalamic-pituitary-adrenal (HPA) axis response correlates with maternally deprived animals' fearful reactions to novel situations (17). In avian species, quality of maternal care has been related to corticosterone (CORT) levels [*Aphelocoma coerulescens*; (18)]. In young chicks of domestic fowl, auditory and olfactory cues from a mother hen have positive effects on learning and memory (19), and stress levels (20). Maternal care in chicks also promotes exploratory behavior (21). Both fearfulness (22) and knowledge about potential threats is communicated from (surrogate) mother hens to chicks (23, 24), suggesting that (aspects of) maternal care may have long-term effects on (among other things) the HPA-axis, including CORT release.

Maternal care, or lack thereof, can also lead to long-term changes in neuroanatomy visible even in adulthood in humans and rodents (25). Previous studies from our group have demonstrated long-lasting effects to maternal care on chicken brain; maternal care to layer chicks in early life caused increased expression of arginine vasotocin (AVT; a vasopressin ortholog found in birds, reptiles and fish) in the medial preoptic area of the hypothalamus (26) and alterations in differences of cell size between the two hemispheres in the hippocampus (27) when brains of adult animals were examined. AVT receptor expression is also modulated by CORT in avians (28), indicating a link between the two systems.

Given the impracticality of housing broody hens with chicks in industrial situations on the one hand, and the desirability of introducing aspects of maternal care to improve welfare and reduce energy use on the other hand, investigation of the effects of the use of dark brooders during rearing on physiological parameters related to stress and welfare is of strong interest. Previously published results from the present group of animals demonstrated strong positive effects of dark brooders on plumage condition, wound reduction and reduction in mortality rates (13). To further examine mechanisms behind these changes, we therefore investigated effects of rearing hens with a dark brooder from hatch through 6 weeks of age on CORT concentrations in feathers and on AVT expression in the hypothalamus at 16 and at 28 weeks of age.

## Materials and Methods

All procedures involving animals were in accordance with the Danish Ministry of Justice Law No. 382 (10 June 1987) and Acts 333 (19 May 1990), 726 (9 September 1993) and 1016 (12 December 2001). This study is part of a larger project that also evaluated the effects of brooders on welfare, including behavior, fear, injurious pecking damage, mortality and production parameters (7, 13).

### Animals

The chickens used in this study were non-beak trimmed Isa Warren layers, obtained when 1 day old from TopÆg Aps, Viborg, Denmark.

### Housing

Animals were housed at the animal housing facilities at Aarhus University, Foulum, in Denmark. Upon arrival groups of 100–103 individuals were placed in 22 pens, each measuring 4 × 2 × 2 m (Length × Width × Height). When 16 weeks old the group sizes were reduced to 50 birds/group. There were four different brooder treatments (each consisting of four replicate groups) differing in size of the available area under the brooder per chick (54 or 72 cm^2^) and in whether the brooder was raised at regular intervals [for more details see (13)]. Due to the time consuming and costly nature of the procedures applied in the present study, only one of the brooder treatment, i.e., four treatment groups, and only five of the six control groups were included.

In the dark brooder treatment selected, one water-heated dark brooder (Jyden Bur A/S) of 120 × 60 cm was present in each of the pens. The dark brooders were 15 cm from the ground in the “lowered” position such that the chicks could access a warm and dark area under the brooders; the chicks could all fit under the brooder and were regularly observed to do so. Every 4 h on days 0–4, the dark brooders were lifted 140 cm from the ground for 10 min to mimic the behavior of a mother hen when initiating an active period. After day 4 of age, the brooders remained in the lowered position. The brooders were then permanently raised to 200 cm above the ground when the chickens reached 41 days of age. Throughout the time that the dark brooders were present in the pens, the dark brooders were used as a primary heat source, with the temperature under the brooder being 34°C for the first 3 days, then lowered a half-degree each day until 20°C was reached on day 30. The room temperature in the dark brooder group was kept at 24°C on day 0 and 1, 22°C on day 3 and 4 and then kept constant at 20°C. The control groups were housed in an adjacent identical room where the room temperature was kept at the same temperature schedule as used for the temperature under the brooders. See Table 1 for a schematic representation of temperatures for both groups. In order to minimize pen-effect, all groups were reallocated over the 22 pens at day 44 of age.

**Table 1:** Schematic overview of the room temperatures used for control and dark brooder groups and the temperature under the dark brooders.

**Day**	**1**	**3**	**4**	**5**	**7**	**10**	**12**	**14**	**16**	**18**	**20**	**22**	**24**	**26**	**28**	**30**	**42**
T_R_ brooder	24	22	22	20	20	20	20	20	20	20	20	20	20	20	20	20	20
T_R_ control	34	33	33	32	31	30	29	28	27	26	25	24	23	22	21	20	20
T_UB_ brooder	34	33	33	32	31	30	29	28	27	26	25	24	23	22	21	20	20

*All values are in degrees Celsius. T_R_, Room Temperature; T_UB_, Temperature under the brooder*.

### Experimental Animals

For the histological and feather CORT investigations 2–3 animals from each of the control groups and three animals from each of the brooder groups were selected. This was done both at 16 w of age (113–114 d of age, just before group size reduction) and again at 28 w (198–199 d) of age, giving a total of 48 animals for the present experiment (*N* = 12 per treatment per age). Animals were selected randomly but were required to fall within the 25th and 75th percentile with regards to body weight, as determined by weighing another group of animals from the same strain, age and supplier housed in the same facilities. None of the pullets had come into lay at 16 weeks of age. In addition to being within the 25th and 75th percentile with regards to body weight, the hens selected at 28 weeks of age also had a comb of a normal size and color, indicating that they were in lay.

### Feather Collection and Brain Dissection

The selected animals were sacrificed by cervical dislocation. Primary wing feathers 2 and 8 were plucked from both wings from each chicken, protected from light with aluminum foil and stored at room temperature until processing. Brains were dissected then immersion fixed in 4% paraformaldehyde. After 24 h, brains were transferred to 70% ethanol and stored at 4°C until sectioning.

### Brain Sectioning and Immunohistochemistry

Before sectioning, the chicken brains were embedded in gelatin and stored in a 30% sucrose solution at 4°C. The gelatin embedded brains were sliced according to the chicken atlas (29), with sections collected from rostral to plate 13 until caudal to plate 15 using a vibratoom (Leica VT1200S) set at 40 μm. Sections were collected in four series, thus with 160 μm between each section and stored in tubes of 0.12 M phosphate buffer saline (PBS) and 0.1% sodium azide at 4°C.

#### Single-Label Immunohistochemistry

Procedures for AVT immunohistochemistry (IHC) were as in Hewlett at al. (26). Briefly, IHC was done using a Vector labs Elite ABC kit (Brunschwig Chemie, Amsterdam) on free-floating sections. Unless otherwise stated, all steps were done at room temperature and by placing the sections on a low speed shaker to help evenly expose the sections to the solutions. Sections were placed into 0.12 M PBS for 10 min. Subsequent washing steps were in 0.05 M tris-buffered saline (TBS) for 5 min and repeated three times. Sections were then washed before incubation in 0.3% H_2_O_2_ in methanol for 10 min to remove endogenous peroxidase. After another washing step, the sections were incubated for 1 h in 5% normal goat serum (NGS) blocking buffer in 0.03% triton-X100 in TBS (TBST). Excess buffer was removed and the slides incubated in primary antiserum for AVT (rabbit anti-AVT 1:2,500, kind gift from Prof. S. Blähser) in 1% NGS-TBST blocking buffer for 1 h at room temperature and then at 4°C for 20 h. The following day, sections were washed three times in TBST before incubation for 1 h in secondary antiserum (goat anti-rabbit IgG 1:200, DAKO, Denmark) in 1% NGS-TBST blocking buffer. After washing, sections were incubated in 1% avidin/biotin solution (Vectastain ABC-Elite kit) in TBS for 45 min. Sections were then washed before being stained with 3,3'-Diaminobenzidine (DAB, Sigma) with 0.02% H_2_O_2_ peroxidase in TBS for 120 s, then washed again. Finally, sections were mounted onto SuperFrostplus® slides, allowed to dry overnight, processed through a clearing and dehydration series of ethanol and xylene to remove lipids and water, and cover-slipped using DePeX (Serva Electrophoresis, Heidelberg).

#### Antiserum Specificity

To prevent cross-reaction to the related peptide mesotocin, the AVT specific rabbit serum was absorbed to oxytocin (the mammalian equivalent of mesotocin) coupled to CNBR-activated Sepahrose4B according to the manufacturer's protocol (GE Healthcare, the Netherlands) and the non-binding fraction of the antiserum was used. In addition, the specificity of the antiserum specific for AVT in chicken tissue was demonstrated by replacing the primary antibody with normal rabbit IgG as a control for non-specific interaction. This IHC study was possible through generous contribution of Prof. S. Blähser of pure Rabbit anti-AVT serum.

#### AVT-Positive Neuron Counting

All suitable sections obtained from the staining procedure were subsequently counted according to the atlas of the chicken brain by Puelles et al. (29). All of the slides which were undamaged and in the area of interest were counted, which equaled 3–9 slides per animal. AVT-positive neurons were counted in the following hypothalamic areas: medial preoptic area (MPA); medial preoptic nucleus (MPO); paraventricular nucleus, rostral part (prepeduncular hypothalamus) (PaRO); lateral preoptic area (LPO); anteromedial preoptic nucleus (AMPO); supraoptic nucleus (SO). All slides were counted manually and then averaged among the total slides used per chicken. If a region was not present in all the slides, then that region was averaged over the slides in which that area was present. All counting was done using a Jenaval microscope under the magnification (100×) and (200×). The final statistics were conducted on observations from a single observer, but at the beginning and the end of measuring, counting was conducted by two observers to “benchmark” and ensure that the counting had not changed during the course of analysis. Photographs of the slides for reporting were taken using an Olympus BX40 at (40×).

#### CORT Extraction

CORT Extraction from feathers was performed using a modified version of the protocol described in Bortolotti et al. (30), as previously described in von Eugen et al. (31) and Zeinstra et al. (32). Extraction began by cleaning the feathers using aerosol solution of methanol. The calamus and rachis were removed and only the vane region was used for analysis. The feathers were cut in slices of roughly 5 mm^2^ flakes. The feathers were measured in length before and after cutting and then weighed. The extraction itself consisted of sonicating the feathers in a water bath for 30 min and subsequently incubating the flakes in an 80% methanol solution, 5 mL with an additional 1 mL/0.03 g of feather, overnight. The samples were centrifuged the following day at 2,200 g for 10 min at room temperature (20°C). The supernatant was then divided into 4 Eppendorf tubes per feather sample and dried using a vacuum pump (Speedvac, Savant AES 1000-240) at a medium dry rate for 3 h. Dried residue was later reconstituted using the respective buffers from the kit used for analysis.

#### CORT Enzyme Immunoassay (EIA)

CORT EIA was performed using the CORT EIA Kit (Cayman Chemical Company). After buffer, sample and reagent preparation, standards were made according to protocol ranging from 5,000 to 8.2 pg/mL. All reagents, standards, samples and buffers were added to the wells according to a scheme and allowed to incubate for 2 h on a shaker. The plate was then washed 5 times in wash buffer. Right before use, Ellmans reagent was reconstituted and added to the plate and allowed to incubate for 1 h at room temperature in total darkness. The plate was then subsequently read at 405 nm. All values are expressed as pg CORT per mg of feather vane.

The results from the CORT EIA obtained from the Microplate reader (DTX 880, Beckman Coulter) were analyzed using an excel file provided from the company that produced the kit (Cayman Chemical Company). The excel results were then statistically analyzed using SAS.

### Statistical Analysis

The assumption of normality was assessed using the Kolmogorov-Smirnov test (PROC UNIVARIATE, SAS Inst. Inc., Cary, NC). For the IHC results, it was not possible to achieve a normal distribution to both conditions using transformations, thus a Friedman's two-way non-parametric analysis was performed on the ranked values with age and treatment (brooder vs. control) as main effects, and age × treatment interaction.

Visual inspection of the data indicated a possible outlier in the corticosterone data from the control group; this was statistically confirmed with a Grubbs' test, also called the ESD method (extreme studentized deviate), thus data from one control animal were removed from this analysis. The CORT levels expressed in pg/mg feather were normally distributed following exclusion of this outlier, thus ANOVA analysis was performed. A repeated measures ANOVA was conducted with feather (2 and 8) as repeated measure, and with the main effects age (16 weeks vs. 28 weeks) and treatment (brooder vs. control). The experimental unit was individual animal.

## Results

### Hypothalamic AVT Levels

**Figure 1:**
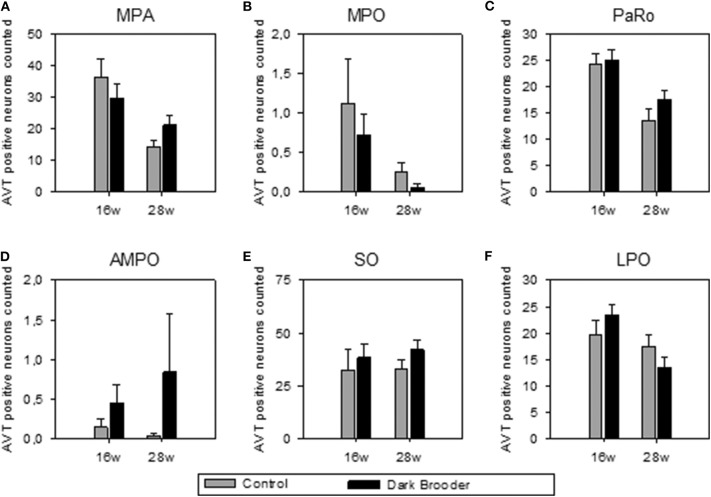
Number of AVT-positive neurons counted per region of interest (mean ± SEM). **(A)** MPA, medial preoptic area; **(B)** MPO, medial preoptic nucleus; **(C)** PaRO, paraventricular nucleus, rostral part (prepeduncular hypothalamus); **(D)** AMPO, anteromedial preoptic nucleus; **(E)** SO, supraoptic nucleus; **(F)** LPO, lateral preoptic area. 16w, 16 weeks of age; 28w, 28 weeks of age.

The number of AVT-positive neurons in the MPA, MPO, PaRO and LPO was strongly influenced by age, with higher numbers of AVT-positive neurons in all four areas in the 16 w old animals compared to the 28 w old animals (see Figure 1, Table 2). Trends to effects of brooder raising were found in both AMPO and SO, with dark brooder reared animals showing higher mean counts of AVT-positive neurons in both areas. No interactions between brooder raising and age were observed.

**Table 2:** Statistical results of AVT-positive neuron count comparisons (Friedman's Two-Way Nonparametric ANOVA).

	**Treatment**		**Age**		**Treatment × Age**	
**Brain area**	***F***	***p***	***F***	***p***	***F***	***p***
MPA	0.73	0.40	**13.76**	**<0.001**	2.04	0.16
MPO	0.76	0.39	**18.03**	**<0.0005**	1.73	0.20
PaRO	1.51	0.23	**19.78**	**<0.0001**	0.34	0.56
AMPO	*0.93*	*0.09*	2.64	0.11	0.13	0.72
SO	*3.74*	*0.06*	1.06	0.31	0.01	0.92
LPO	0.01	0.93	**6.23**	**<0.05**	1.81	0.19

*Statistically significant results printed in bold, statistical trends printed in italics. MPA, medial preoptic area; MPO, medial preoptic nucleus; PaRO, paraventricular nucleus, rostral part (prepeducular hypothalamus); AMPO, anteromedial preoptic nucleus; SO, supraoptic nucleus; LPO, lateral preoptic area. Treatment = brooder vs. no brooder, Age = 16 weeks vs. 28 weeks of age*.

### Feather CORT

**Figure 2:**
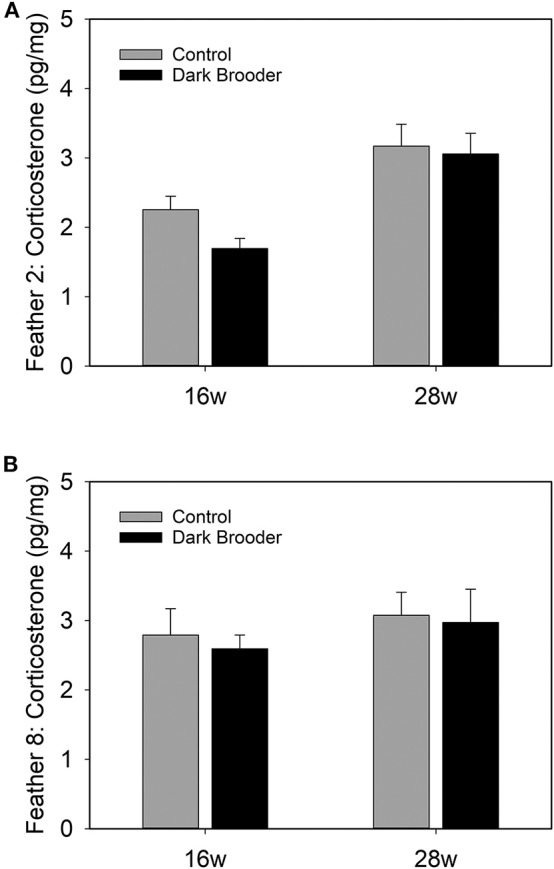
Bars indicate concentrations of CORT in pg per mg of feather vane measured in primary wing feather 2 **(A)** and primary wing feather 8 **(B)** from hens at 16 weeks (16 w) and 28 weeks (28 w) of age. Bars indicate average, error bars indicate SEM.

CORT levels were higher in primary wing feathers from 28 week old hens than in those from 16 week hens (Figure 2). This effect was seen in both feathers 2 and feather 8, and a main effect of age was confirmed statistically (see Table 3). No main effects of rearing with a brooder or interactions between effects were found on CORT levels in the feathers sampled.

**Table 3:** Statistical results of CORT in primary wing feathers (pg CORT/mg feather, repeated measures ANOVA).

	***F***	***p***
**Weeks of age**	**4.13**	**0.04**
Treatment	2.11	0.15
Feather	1.03	0.31
Weeks of age × treatment	2.13	0.15
Feather × weeks of age	0.32	0.58
Feather × treatment	0.04	0.85
Feather × weeks of age × treatment	0.44	0.50

*Statistically significant results printed in bold. Treatment = brooder vs. no brooder, feather = feather 2 vs. 8, weeks of age = 16 weeks vs. 28 weeks of age*.

## Discussion

Age had an effect on both AVT expression in the hypothalamic areas MPA, MPO, PaRO and LPO, and CORT concentrations in feathers, with CORT levels higher in older animals and the number of AVT-positive neurons higher in younger animals. Rearing with brooders did not significantly alter CORT or AVT levels, though brooder rearing did produce a trend to increased AVT in AMPO and SO.

### AVT Expression in Relation to Dark Brooders and Age

The AVT-positive immunoreactive neuron distribution observed in the present study is comparable to that previously reported in chicken, i.e., from our lab by Hewlett et al. (26), and previously by Viglietti-Panzica (33) and Tennyson et al. (34). Broadly similar distributions have been reported in other avian species, such as starling, duck and Japanese quail by Bons (35), Panzica et al. (36) and Goossens et al. (37), and in the dark-eyed junco by Panzica et al. [*Junco hyemalis*, (38)]. Although brooder rearing did not significantly alter the number of AVT-positive neurons, trends to increased numbers of AVT-immunopositive neurons were seen in the SO and the AMPO. This alteration in AVT cannot directly be linked to positive or negative welfare states; given that both excitatory and inhibitory transmitters are localized in the hypothalamus, sometimes even colocalizing with neuropeptides within a single neuron (39). However, the trend to effect indicates a potential link- be it positive or negative- with the physiological functions in which the SO and AMPO play a role.

There is a wide body of evidence linking the SO to physiological functions in avians, specifically to osmotic and hemodynamic regulation. Osmotic stress and hypotensic stress increase AVT expression in the chicken SO (40–42). Osmotic stress also increases c-fos expression in AVT-positive neurons in the SO in chicken, zebrafinch, ring dove, and Japanese quail (43), and increases neuron size in the SO in quail (44, 45). It is interesting in this context that animals reared with brooders spent less time drinking during the first 4 days of life, probably due to a more efficient thermoregulation resulting in less respiratory evaporation (13).

The specific role of the AMPO has been less well-described. Lesions in the AMPO in rats have been linked to disruptions of the pre-ovulatory LH surge and reduction in prolactin surge (46, 47). The AMPO also undergoes changes in rat in relation to parity (48). The mammalian equivalent to AVT, vasopressin, has been shown to stimulate an LH surge when injected directly into the preoptic area (including the AMPO) (49). In all, this indicates a potential link between increased AVT in the AMPO and ovulation, which could mean that dark brooders may be beneficial to ovulation and thus potentially to egg laying.

Age-dependent decreases in AVT were observed in the MPA, MPO, PaRO, and LPO areas of the hypothalamus. The age-related expression of AVT may be related to sexual development and lay onset in the chickens in the present study. The MPO has been linked to nest-exploration behavior in starlings, with higher estradiol, higher preproenkephalin (PENK) mRNA and lower levels of D1 and D2 dopamine receptor mRNA in the MPO in female starlings which had explored a nest than those which had not (50). Onset of laying in Isa Warren hens is generally around 19 weeks of age, with peak production at 26 weeks of age. The 16-week hens in the current study would have been just prior to the start of laying, while the 28 week hens are at peak production levels. AVT expression in ovaries has been strongly linked to oviposition in avian species (51–53). Previous studies in quail examining age-dependency of AVT expression in ovaries found no evidence of AVT expression in ovaries at 4 weeks of age (just prior to lay onset), high expression at 12 weeks of age (at peak egg production), and lowered expression at 78 weeks (at low egg production levels) (54), underlining the role of AVT in sexual maturity in avian species.

Interestingly, all of the areas examined showed either significant effects of age or a trend to an effect of brooder rearing, but not both. No interactions were seen between the two main effects. This indicates a quite strict topographical separation of effects in the areas examined.

### CORT Levels in Feathers Increase From 16 to 28 Weeks of Age

In the current study, higher levels of CORT were found in feathers taken from 28 week old hens compared to 16 week old pullets. A major event that takes place between these two ages is onset of laying, i.e., the transition from pullet to a sexually mature hen. It is not known exactly how long CORT is stored in feathers, or how long it takes for circulating CORT to accumulate in feathers, but it is a reasonable assumption that an increase observed in feathers at a later age is a reflection of higher circulating CORT levels at later ages. No effect of brooder rearing was seen on CORT levels in feathers. This may indicate that either effects of brooders are too subtle to be measured in the long-term accumulation of CORT in feathers, or, alternatively, that the use of brooders does not directly affect the HPA-axis. Further research in this area is warranted.

The age-dependent increase in feather CORT observed may be related to onset of laying in the hens in the current study. In avian species, stress is strongly linked to delay in lay onset [reviewed in (55)]. Direct manipulation of the HPA-axis also impacts lay onset: oral administration of CORT for three seven-day periods in drinking water before onset of laying, delays lay onset in layer hens (56). The HPA-axis, and specifically cortisol, has also been linked to the onset of sexual maturity in other species. In humans, an increase in urinary cortisol is seen in perimenarche that is correlated to both gynecological and chorological age (57). Increased salivary cortisol response to the Leiden Public Speaking Task as a stressor correlated with self-reported pubertal development in humans (58). In rats, an increased cortisol response to an acute stressor is seen in a series of studies examining pre-pubertal rats compared to adult rats [reviewed in (59)]. In all, an increase in cortisol or CORT around the timepoint of sexual maturity is frequently seen in various species, and may explain the age-dependent feather corticosterone increase in the present study.

### Conclusions

Age was found to affect the expression of AVT in the hypothalamus of chickens, and an age-dependent increase in CORT was seen in feathers of the hens. This information aids in further understanding of the development of chickens around puberty. The use of brooders tended to affect AVT expression in some areas of the hypothalamus. The lack of effect of dark brooders on CORT in feathers is at the least an indication that the use of dark brooders is not stressful; given previously shown positive benefits of dark brooders, e.g., prevention of injurious pecking, reduced fearfulness and lower mortality, this pleads for the use of dark brooders in on-farm situations.

## Data Availability Statement

The datasets generated for this study are available on request to the corresponding author.

## Ethics Statement

All procedures involving animals were in accordance with the Danish Ministry of Justice Law No. 382 (10 June 1987) and Acts 333 (19 May 1990), 726 (9 September 1993) and 1016 (12 December 2001). This study is part of a larger project that also evaluated the effects of brooders on welfare, including behavior, fear, injurious pecking damage, mortality and production parameters (7, 13).

## Author Contributions

AR conceived and designed the study with RN. RN and AR drafted the manuscript with help from EZ. EZ and AD performed laboratory work, analysis of stained slides, and statistical analysis.

### Conflict of Interest

The authors declare that the research was conducted in the absence of any commercial or financial relationships that could be construed as a potential conflict of interest.
